# The Governance of Standard-Setting to Improve Health

**Published:** 2010-10-15

**Authors:** Daniel M. Fox

**Affiliations:** Milbank Memorial Fund

## Abstract

This article describes recent events in the governance of standard-setting for 2 areas of US health policy — states' decisions about which prescription drugs to cover under Medicaid and other public programs and making health an aspect of foreign policy — and whether these events offer lessons for policy making. In prescription drug coverage, methodologic advances in research that evaluates health services and the politics of restraining the rate of growth in health expenditures enabled policy makers in most states to establish new public processes for assessing and applying evidence about the effectiveness of competing drugs. Their counterparts in foreign policy, in contrast, made few changes in existing processes for choosing which interventions to support. The history of governance in each area of policy making for health explains the selection of standards to evaluate evidence about interventions and whether and how to use this evidence to guide policy.

## Introduction

Government leaders at every level choose among alternative policies mainly as a result of governance. Researchers in the policy sciences (eg, history, politics, economics, law) describe governance as encompassing the complex relationships among people and organizations that influence the making and implementing of policy. Understanding governance requires analysis of the authority and accountability embodied in constitutions, laws, and regulations; the politics of professional, commercial, and advocacy groups; and the shaping of public opinion. Moreover, ideas and beliefs — some contested, others consensual — influence the governance of each area of policy. In sum, governance is the source of the "power to make, the willingness to obey, and the decisions to contest rules and commands" (1).

This article describes, compares, and seeks lessons from the effects on standard-setting of recent changes in the governance of health care policy in the states and of health as an aspect of American foreign policy. During the past decade, almost all of the states established public processes to set standards for evaluating research findings on the effectiveness of pharmaceutical drugs, for adjudicating competing claims about the strength of the evidence for these findings, and for advising about or, in some jurisdictions, recommending policy. In contrast, the events that raised the priority accorded to health as an aspect of foreign policy did not establish new processes that set standards for how the best available evidence would inform policy. As a result, the conventional governance of foreign policy set standards for which determinants of health to address and with what interventions.

## History of the Governance of Population Health

### Governance and the delegation of authority

Until recently, the governance of most countries' jurisdictions resulted in the authority for setting standards for health policy being split among different influential groups. Public officials set standards for investigating, measuring, and, if possible, acting to reduce the incidence and prevalence of disease and improve the safety of patients in clinical facilities. In each country, governance determined the influence of the best available research and lobbying by commercial, professional, and reformist interest groups on these standards.

Governance in most countries resulted in authority for science being delegated to communities of researchers. Researchers usually dominated the prioritization of subjects for investigation and set standards for methodology and evidence. They governed science through professional associations, national academies and colleges, universities, foundations, and government funding agencies.

Governance also resulted in authority being delegated to the health professions. For centuries, physicians have had legal authority to license, certify, credential, and discipline their colleagues. As a result of this authority, they acquired substantial autonomy beyond what had been legally granted to them to set and enforce standards for care. Physicians tenaciously protected this autonomy when, during the 20th century, governments delegated more limited authority to other health professions to license and discipline their members.

However, governance could not divide authority to make and implement policy to address determinants of health that involved physical infrastructure, personal behavior, and socioeconomic conditions. For example, since the 19th century, coalitions in the United States and other countries supported the allocation of considerable tax revenue for sewerage and the chlorination and filtration of water. By the early 20th century, investment in technologies to produce clean water was "responsible for nearly half the total mortality reduction, three quarters of the infant mortality reduction, and nearly two thirds of the child mortality reduction [in] major American cities" (2).

### Innovations in governance

Other innovations in public health policy occurred as a result of governance that involved public agencies, the medical profession, and leaders of business, philanthropy, and labor. For example, international collaboration among researchers and public officials to define diseases in order to report and quantify cases began in the 1850s. By the end of that decade, William Farr, a British health official, had devised a "model healthy population to serve as a standard" for calculating excess mortality among health districts (3). By the 1980s this concept, elaborated, had become the basis of the *European Community Atlas of "Avoidable Death"* (4).

By allocating resources to address other determinants of population health, governance facilitated the implementation of health care policy. Beginning in the 1850s, for instance, William Farr collaborated with Florence Nightingale in achieving policy to measure excess deaths in public and charitable hospitals. Then they acquired resources to evaluate interventions to reduce excess mortality by intervening in both the care of patients and the management of hospital environments (5). Efforts continue to persuade policy makers to link interventions with individuals and with populations. For example, a recent US study of avoidable deaths found that "health improvement requires investment in . . . health care, behavioral change, and socioeconomic factors" (6).

### Addressing multiple determinants of health in governance

Governance also has been mobilized to address multiple determinants of health. One of the earliest examples of this mobilization occurred in New York City in the 1890s when public health officials proposed mandatory reporting of tuberculosis, which the medical profession strongly opposed. Then the city's political machine, Tammany Hall, along with leaders of business and philanthropy who usually opposed Tammany, endorsed mandatory reporting (7). Another example of the mobilization of governance to address multiple determinants of health occurred in many low- and middle-income countries from the 1920s through the 1960s. Public officials in these countries, often collaborating with leaders in business, labor, religion, and philanthropy, prioritized investment in raising standards for education and public health rather than for health care (8).

Governance in industrial countries frequently results in the prioritization of determinants of health other than care during crises. Until the mid-19th century, for instance, hunger and its effects were not problems of governance. Prevailing belief ascribed hunger to individual misbehavior or inexorable natural forces. Governance then redefined hunger as a problem caused by economic, social, and political circumstances. By the 1920s, scientific advances distinguished starvation from malnutrition, and policy emerged to address both conditions. During World War II, a British official described the effects of public, civic, and private activities to prevent starvation and malnutrition. He reported that the "people of this country are actually better fed today from the point of view of health than they were before the war" (9).

In each of the examples above, participants in governance had incentives to address determinants of population health. Healthier voters enhanced Tammany's political capital and were more productive employees. Policy makers and their allies in low- and middle-income countries built schools and educated their citizens about managing health risks, in large part because they had fewer resources than their counterparts in industrial countries. The governance of wartime Britain strongly endorsed food policy that maintained a productive workforce and contained class conflict.

Precedents also exist for standards that address multiple determinants of health in the governance of foreign policy. During the 1930s the League of Nations Health Organization promoted science-based standards for nutritional policy, usually collaborating with external scientific, professional, and philanthropic organizations. In the 1950s, leaders of philanthropic foundations and public officials in the United States collaborated to expand the scope of foreign policy to include aid for family planning in low-income countries.

## The Conventional Politics of Setting Standards for Health

In each of these examples, research findings on population health informed governance through conventional political processes. Researchers, physicians and other health professionals, advocates for patients, and lobbyists for commercial interest groups published studies and polemics, informed journalists, testified to legislative committees, visited policy makers, and contributed to their campaigns. Officials of national and subnational governments, multinational public organizations, philanthropies, and advocacy groups issued reports and promoted policies to set and raise standards for health.

Unanticipated consequences of these conventional mechanisms of governance impeded making policy to improve population health. Elected officials have had grounds for skepticism about scientific advice given to them by patients' advocates, workers, members of racial and ethnic minority groups, and even charitable organizations, as well as from lobbyists for commercial and professional organizations. Policy makers have, for instance, often distrusted advice from career scientists within government because these civil servants have frequently collaborated with (and subsequently became employees of) advocacy and industrial organizations that interpreted scientific evidence in ways that promoted their self-interest (10).

## Changes in Governance

### Advances in research and evaluation methods

Despite interest-group lobbying and the skepticism of policy makers, science that met international standards of excellence has frequently been effective in the governance of population health policy. Examples include regulating lead in gasoline and paint, asbestos in building materials, and vinyl chloride as an industrial chemical (11) and limiting exposure to secondhand tobacco smoke in public places and workplaces. In each of these instances, findings from research that was independent of commercial or ideological influence helped government officials persuade colleagues and constituents to support new regulations, even when these policies adversely affected the earnings of corporations and individuals and restricted personal liberty.

Advances in methods for evaluating the effectiveness of health services have influenced governance around the world since the early 1990s. These methods enabled policy makers to challenge assertions about what services to pay for that were based mainly on claims of authority by medical professionals and sometimes on questionable evidence promoted by commercial and advocacy groups. The most prominent example of this influence of research on governance is the methodology of research synthesis and its use to conduct systematic reviews of the effectiveness of prescription drugs, medical devices, care processes, and public health interventions. Authors of systematic reviews who accept international standards exclude the weakest and most biased primary studies and conduct meta-analyses to minimize bias in studies they select for synthesis. The number of systematic reviews published each year in the international literature recently increased from 87 in 1988 to an average of 2,500 in 2005 (12).

Methodologic advances that have increasing influence on governance also occurred in other disciplines in recent decades. New methods for measuring and improving the quality of health care, work that was subsequently labeled quality science, evolved from the study of industrial processes in the general economy and from general and clinical epidemiology. Advances in the methods of economics increased the persuasiveness of cost-effectiveness analysis and created new approaches to studying social well-being and analyzing different forms of organizational governance. Similarly, advances in the methods of political science, sociology, and historical epidemiology generated findings that interest some key participants in governance; for example, quantifying the relationship between changes in health care infrastructure and health status, educational attainment, and even the stability of regimes in low-income countries.

### The new governance of evidence-informed standards

Recent innovations in the governance of health care in most industrial countries are assisting policy makers to counter pressure from interest and advocacy groups in new ways. Policy makers have established organizations — sometimes called agencies, commissions, committees, councils, or institutes, but which will be called *review organizations* hereafter — that commission, conduct, and report on independent research that evaluates interventions. These organizations usually recommend policy or issue guidance that has the force of law. The first review organizations assessed new interventions, especially those involving drugs and devices, but their scope is steadily expanding. Review organizations are led by experts in health research, policy, and clinical practice or appoint such experts to advisory groups (13).

Staff of these organizations often share experience across national and subjurisdictional boundaries. As a result of these exchanges, most of the organizations are applying internationally accepted standards for methods to evaluate drugs, devices, and care processes. Research from one country often supports a report under attack in another.

Review organizations dealing with the governance of health care have antagonists. Manufacturers of drugs and devices, the research and advocacy groups they finance, and some associations of medical specialists frequently challenge public and quasi-public organizations that evaluate health services. These critics often deplore decisions that limit coverage to the most effective interventions. Many insist that analysis of cost-effectiveness masks decisions to ration care.

The frequency and sophistication of these challenges has increased since the 1990s because of the rapid increase in the number of public, quasi-public, and nonprofit organizations that use evidence-based health research to inform their recommendations. This growing use of evidence-based health research followed the advances in methodology summarized above. These advances influenced governance because they coincided with the dismay of many policy makers and employers about increasing expenditures for health care. The first project to use systematic reviews to evaluate an entire area of health services published its results in 1989 (14). The Cochrane Collaboration, organized in 1993, has established an international process for improving the standards and methods of systematic reviews. It also created, enlarged, and sustained an international community of reviewers.

The standards set by most of the review organizations threaten manufacturers and their allies in the supply chain, as well as many researchers, because they address sources of systematic bias in conducting and reporting research. For example, the review organizations' standards for disclosing and avoiding conflict of interest are often higher than those of most universities and funders of primary studies. Many review organizations also require that evidence submitted to them by industry be made publicly available.

Despite considerable opposition, evidence is accumulating that policy created on the basis of the work of organizations that conduct and assess systematic reviews of prescription drugs and other interventions is improving the quality of care and containing growth in spending. The application of science-based regulatory standards shifts market share, often drastically, to the most effective interventions.

Some public review organizations in the United States and other countries also evaluate interventions to prevent disease and address determinants of health other than care. The United States Preventive Services Task Force systematically reviews evidence of effectiveness and issues recommendations. The Guide to Community Preventive Services of the Centers for Disease Control and Prevention commissions systematic reviews of interventions to improve population health but does not recommend policy. The National Institute for Health and Clinical Excellence (NICE) in the United Kingdom has published public health "guidance" based on evidence reviews for interventions that have recently included behavior change, community engagement, social and emotional well-being in primary education, and promoting physical activity. Policy makers have recently asked a few review organizations to recommend the reallocation of resources from ineffective services to address determinants of health other than care. Such public discussion has occurred — and generated controversy in governance — in Australia, England, France, and Spain (15).

The changes in governance that have raised evidentiary standards for policy for health care and population health are a result of the gradual redistribution of power. Redistribution is occurring because of growing agreement on 2 points among many leaders of government, business, the health professions, and the media: 1) that the rate at which spending for health care has been increasing is unsustainable and 2) that much care is ineffective, unnecessary, or harmful. This agreement is reflected in changes in governance that are mitigating political barriers to higher evidentiary standards for the coverage of health services (eg, the sections on comparative effectiveness research in Patient Protection and Affordable Care Act of 2010 in the United States) (16,17). These barriers are, however, still daunting.

## Standards for Health in the Governance of Foreign Affairs

Improving health has become a funded rather than symbolic goal of foreign and national security policy since the late 1990s. The US Central Intelligence Agency reported in 1998 that high infant mortality was a significant predictor of the failure of states. During the second Clinton Administration, the National Security Council for the first time assigned a staff member to address issues in global health. In 2001, a new secretary of state, Colin Powell, appointed the first assistant secretary of state for health. Ambassadors rather than aid officials in Washington and low-income countries administered the President's Emergency Program for AIDS Relief (PEPFAR) enacted in 2003. A committee of the Institute of Medicine recommended that the incoming Obama administration "highlight health as a pillar of US foreign policy." The United States and other donor countries increased spending for health by more than 600% during the past 2 decades (18-20).

The salience of health as an aspect of foreign affairs increased without changes in governance as substantial as those that have occurred in decision making for health care. Policy makers for health in foreign affairs and their allies outside government have often refused or been reluctant to apply findings from research on the effectiveness of interventions. Some opposition to applying the findings of independent research is ideological (eg, advocates of abstinence-only programs to prevent HIV infection) or commercial (eg, resistance from pharmaceutical companies to purchasing generic drugs with PEPFAR funds).

Many experts on international health and their allies in government have also resisted applying the best available findings from research. Following are some examples from my experience. A Washington-based nongovernmental organization (NGO) appointed an internationally prominent systematic reviewer as its director of research and then denied him access to its grant funds from the Bill and Melinda Gates Foundation. Leaders of health-related NGOs from many countries opposed a recommendation by a work group of the Council on Foreign Relations that PEPFAR take account of findings from systematic reviews (21). The first administrator of PEPFAR in the US Department of State and the program's chief physician met with the authors of the recommendation but declined to accept it. As a participant in these events, I speculated that this resistance to the best evidence was about protecting territory: for NGO leaders, access to and approval by funders in government and foundations; for PEPFAR officials, to avoid collaborating with and perhaps funding federal agencies that sponsor research that evaluates interventions to improve health.

The World Health Organization (WHO) endorses systematic reviews but has been ambivalent about using them to set standards for policy. WHO's Model Lists of Essential Medicines and its program on maternal and child health rely on reviews published by the Cochrane Collaboration. However, WHO continues to recommend Directly Observed Therapy/Short Course (DOTS) for treating tuberculosis despite trials and systematic reviews that find it is not the most effective intervention (22).

Several countries and private organizations are, however, applying standards in global health similar to those that are becoming conventional in the governance of domestic policy for health care. The chief medical officer of the Department of Health in the United Kingdom, for example, leads a "government-wide global strategy" for health that includes using the research and standard-setting expertise of the National Health Service, the Health Protection Agency, and NICE (23,24). Similarly, leading foundations and multinational organizations in global health evince increased interest in evidence from independent research. The governance of health as an aspect of foreign affairs may be changing.

## Conclusion

The use of evidence from research to set standards and inform policy has had a different history in health care, especially in making decisions about coverage, than in health as an aspect of foreign policy. In health care, findings from research in laboratory, clinical, and community settings have been prominent in governance of the allocation of resources and of accountability for more than a century.

In the governance of foreign policy, in contrast, findings from formal research have almost always been subordinate to ideology, commercial interests, and threats to international and homeland security. Participants in governance often have substantial reasons to subsidize and placate leaders of countries that have dysfunctional health systems. Policy makers for health care, unlike their counterparts in foreign policy, work in the context of high public expectations that interventions will have measurable benefits for people and populations.

Proponents of science-based standards in the governance of both health care and health as a factor in foreign policy have experienced less resistance to establishing such standards for health services than for socioeconomic and behavioral determinants of health. Evidence has accumulated about the effects on health status of alternative policies for income maintenance, education, social services, and the environment. But improving health is hardly ever the highest priority of leading participants in the governance of these areas of policy, at home or in other countries. Calculations of potential net improvement in population health status over time are likely to remain secondary to immediate economic and political concerns.

However, recent research on the economics of governance suggests that it is possible and desirable to make policy that addresses broad determinants of health and to do so for both domestic and foreign policy. In his presidential address to the American Economic Association in 2009, Avinash Dixit described the benefits of governance that promotes well-being in a country or region. Such governance "enabl[es] the growth of income and globaliz[es] the enlargement and stability of the middle class." These benefits justify higher standards for population health to inform "collective action" in the "provision of public goods and the control of public 'bads'" (25). Other economists argue that effective incentives for such collective goods exist "outside the standard private goods model" (26).

Moreover, evidence exists that policy has improved population health indirectly, thus avoiding some resistance to making changes in governance to set higher standards for interventions. For example, strong evidence exists that population health in industrial countries improved since the early 19th century, mainly as a result of increased public spending for health, housing, and social services combined with taxes that encouraged capital investment and, by taxing consumption, discouraged behavior linked to poor health and premature death (27).

The history of governance in each of the areas of policy discussed in this article offers lessons for improving population health. The lesson from the governance of health care is that governance can be politically feasible for policy makers to establish science-based standards for policy and create organizations to conduct and assess research effectively. The lesson from the governance of foreign policy is that it can contribute to improving health even when it rejects standards on the basis of the best available evidence. The broadest lesson from the analysis in this article is that governance, in all its complexity, is the principal determinant of policy.
